# Bidirectional and Stepwise Rotation of Cells and Particles Using Induced Charge Electroosmosis Vortexes

**DOI:** 10.3390/bios14030112

**Published:** 2024-02-20

**Authors:** Shaoxi Wang, Zhexin Zhang, Xun Ma, Yuanbo Yue, Kemu Li, Yingqi Meng, Yupan Wu

**Affiliations:** 1 School of Microelectronics, Northwestern Polytechnical University, Xi’an 710072, China; shxwang@nwpu.edu.cn (S.W.); zhexinzhang@mail.nwpu.edu.cn (Z.Z.); mxgie@mail.nwpu.edu.cn (X.M.); likemu@mail.nwpu.edu.cn (K.L.); wyphit@hit.edu.cn (Y.M.); 2 State Key Laboratory of Analog and Mixed-Signal VLSI, Institute of Microelectronics, University of Macau, Macau, China; 3 Faculty of Science and Technology, University of Macau, Macau, China; 4 Research & Development Institute, Northwestern Polytechnical University, Shenzhen 518000, China; 5 Yangtze River Delta Research Institute, Northwestern Polytechnical University, Taicang 215400, China

**Keywords:** controlled rotation, microfluidics, induced charge electroosmosis, dielectrophoresis

## Abstract

The rotation of cells is of significant importance in various applications including bioimaging, biophysical analysis and microsurgery. Current methods usually require complicated fabrication processes. Herein, we proposed an induced charged electroosmosis (ICEO) based on a chip manipulation method for rotating cells. Under an AC electric field, symmetric ICEO flow microvortexes formed above the electrode surface can be used to trap and rotate cells. We have discussed the impact of ICEO and dielectrophoresis (DEP) under the experimental conditions. The capabilities of our method have been tested by investigating the precise rotation of yeast cells and K562 cells in a controllable manner. By adjusting the position of cells, the rotation direction can be changed based on the asymmetric ICEO microvortexes via applying a gate voltage to the gate electrode. Additionally, by applying a pulsed signal instead of a continuous signal, we can also precisely and flexibly rotate cells in a stepwise way. Our ICEO-based rotational manipulation method is an easy to use, biocompatible and low-cost technique, allowing rotation regardless of optical, magnetic or acoustic properties of the sample.

## 1. Introduction

Precise manipulation of cells and particles including transportation, trapping and rotation is an essential requirement in biotechnology [1,2,3,4,5,6]. Among them, rotating is an important capacity utilized in various fields [7], such as single-cell analysis [8], cell imaging [1,9], organism studies, drug discovery and cell microsurgery including cell nuclear transfer [10] and cell injection [11]. In contrast to plane rotation of cells, 3D rotational manipulation can reveal more details of cells, which is useful in cell imaging, cell analysis and cell screening [12].

Although many approaches [12,13] have been proposed for the rotational manipulation of particles and cells, 3D rotation of cells is more challenging. Manual rotation using a micropipette tip to rotate the cells is usually used in laboratories. However, this method has several disadvantages including low efficiency, poor precision and inconsistent performance. A variety of microfluidic methods have been exploited to generate 3D rotation including stepper motors [14], predefined microchannel geometries [15], optical means [16,17], magnetic means [2,18], electrical means [19,20] and acoustic means [21,22,23,24]. The stepper motor approaches can manipulate particles and cells rapidly and precisely, but they need complex devices and are not easily integrated with microfluidic chips. The predefining microchannel geometry approaches are easy to implement, yet they lack accuracy. For optical means, their operations require a high-power laser which is not only expensive but also harmful for cells. Cells can be rotated by magnetic means by embedding magnetic nanoparticles into them which are complicated and may affect the cell viability. Recently, the acoustic approaches have been studied as a powerful tool for manipulating cells. Acoustic streaming flow [24], radiation torque [25] and the surface acoustic wave [26] can be used to rotate samples. Nelson et al. combined microindentation with an acoustically driven, bubble-based device for noninvasive trapping and 3D characterization of objects by driving acoustic bubbles to generate microstreaming flows and rotate samples [22,23]. The limitations of these techniques are that they can only rotate samples unidirectionally near the bubbles and the bubbles are not stable. In addition, the chips are expensive to fabricate and their operations require high voltage amplitudes. Han et al. [27] reported an electrorotation method for measuring the dielectric properties of cells using a three-dimensional octode. Yasukawa et al. [28] developed a simultaneous electrorotation device for monitoring the rotation rate of multiple single cells upon chemical stimulation. Using electrorotation torque [20], 3D rotational manipulation can be achieved in a microchamber, but it requires fabrication of a complex chip using a 3D electrode. Puttaswamy et al. [29] employed a simple method to fabricate a chip with 3D electrodes. By using this chip, they could trap single cells and cell clusters and rotate them stably. The above electric methods for cell rotation are based on dielectrophoresis (DEP), which is related to the electrical properties of cells.

**Figure 1:**
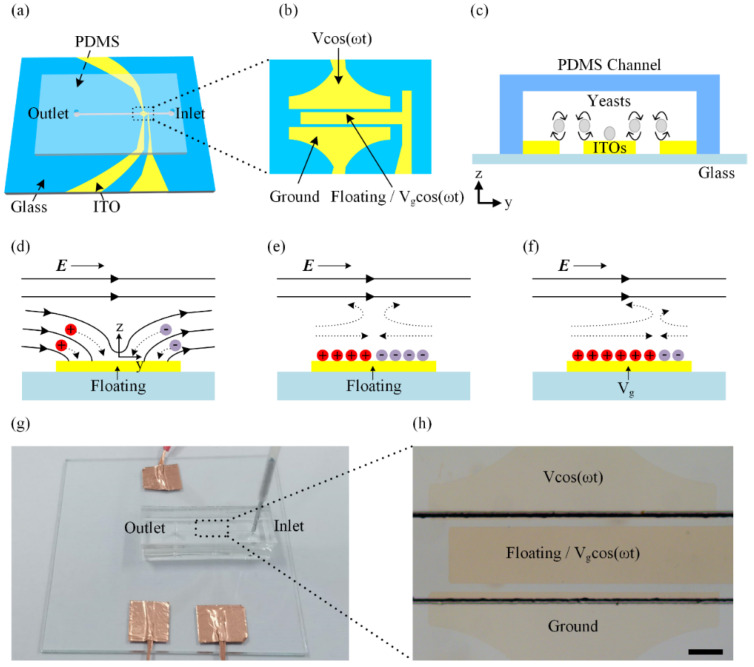
(**a**) A 3D schematic of the device. (**b**) The top view of the ITO electrodes under the main channel. (**c**) A schematic of the experimental setup. The illustration of the basic physics behind ICEO: (**d**) as soon as the electric field is applied, the electric field lines perpendicularly intersect the center electrode at the beginning. (**e**) Then, ions in solution are driven to the center electrode. When the double layer is fully formed, the electric field will be screened from the center electrode and paralleled to the surface of the electrode. The ions in the double layer will be driven to generate slip velocity and two counterrotating vortexes. (**f**) When the center electrode is energized with Vg (Vg < V/2), a positively charged double layer forms, which drives the fluid away towards the grounded electrode and builds asymmetric vortexes. (**g**) Photograph of the assembled device. (**h**) A microscopy image of the black dashed line area in (**g**) showing the main channel and the ITO electrodes. Scale bar, 200 μm.

Induced charge electroosmosis (ICEO) [30,31,32,33,34] is a phenomenon of the nonlinear electroosmotic slip that occurs when an electric field acts on the ionic charge it induces around a polarizable surface. ICEO has received extensive attention due to its notable feature of microflow generation [35,36,37,38,39,40]. The vortexes generated by ICEO have been widely used for cell trapping and microfluid mixing [40]. To achieve cell rotation, cells need to be trapped in advance. A variety of methods have been developed in cell trapping [41,42,43], including optical [44,45,46,47,48,49], acoustic, magnetic [50] and electric techniques [51,52,53]. But optical and acoustic traps always attract the cells to the point of highest energy intensity. Herein, we exploited the ICEO flow around a bipolar electrode that achieves 3D rotational manipulation at different locations in the channel by controlling the applied signal as shown in Figure 1. It can rotate PS microbeads, yeast cells and K562 cells at the bipolar electrode. Our method relies on vortexes generated by electroosmotic slip to 3D rotate cells. Compared with the current electrical methods, the 3D rotation in our method is not related to the electrical properties of cells. The rotation speeds of samples can be changed flexibly by adjusting the applied signals. Additionally, cells can be rotated in a continuous or stepwise fashion to achieve precise control of cell behavior. By applying a gate voltage on the bipolar electrode, cells can be moved from the bottom edge of the floating electrode to the top edge which facilitates rotating cells in a bidirectional way and reducing the applied voltage. This proposed approach is easy to fabricate, more precise, energy saving, biocompatible and controllable, with great compatibility with other chips, making it easier to scale into a versatile tool for cell and microorganism research.

## 2. Theory Background

A schematic of our device is shown in Figure 1a. The device includes a straight PDMS microchannel and a set of electrodes patterned on an indium tin oxide (ITO) glass. The details of the ITO electrodes are shown in Figure 1b. The center electrode is placed in the middle of the main channel, denoted as the gate electrode, and the other electrodes are the driving electrodes. When the driving electrodes are excited by an AC signal and the center electrode is floating, an ICEO flow is generated to produce vortexes in the PDMS channel which can rotate yeast cells in different directions (Figure 1c).

The schematic of ICEO principle is shown in Figure 1d, e and f. The governing equations for the coupled electric potential distribution and flow field can be found in the SI. According to (S1), assuming electrolytes with a constant conductivity σ, the electric potential in the fluid bulk can be achieved. We assume the compact Stern layer as a capacitor connected in tandem to the diffuse layer capacitor. In this case, the total capacitance of the induced double layer (IDL) is:

(1) $$C_{0}=\frac{C_{S}C_{D}}{C_{S}+C_{D}}=\frac{C_{D}}{1+\delta }$$

where $C_{D}=\varepsilon /\lambda_{D}$ is the capacitance of the diffuse layer, $C_{S}$ is the capacitance of the Stern layer, $\delta =C_{D}/C_{S}$ is the ratio of the diffuse layer to the Stern layer capacitance and $\lambda_{D}=\sqrt{D\varepsilon /\sigma }$ is the Debye screening length. $D=2\times 10^{-9}\;m^{2}s^{-1}$ is the bulk diffusivity and $\varepsilon =7.08\times 10^{-10}\;Fm^{-1}$ is the permittivity.

The current from the bulk charges the induced double layer. For the diffuse layer, we can obtain a surface conservation equation:

(2) $$C_{0}\frac{d\Psi_{0}}{dt}=-\sigma \hat{n}\cdot \nabla \phi =\sigma E_{n}$$

where $\Psi_{0}$ is the induced zeta potential, n is the unit normal vector pointing from the electrode into the bulk and ϕ is the bulk potential, $E_{n}$ is the normal component of the electric field.

The boundary condition at the insulating surface becomes ∂ϕ/∂y=0. When the center electrode is floating, using complex amplitudes and the Helmholtz–Smoluchowski equation we can find the ICEO slip velocity on the surface of the center electrode in (S4). When an AC signal is applied to the center electrode, we assume the phase gap is zero and the expression of the ICEO slip velocity is (S6). The slip in this situation is called fixed potential ICEO. Under this circumstance, the potential of the center electrode is coupled to the external circuit and acts like a driving electrode.

The DEP force [54] will act on the particles and cells in solution when an AC signal is applied. If particles and cells are assumed as spherical, the time-average DEP force acting on them can be written as:

(3) $$<F_{DEP}>\;=\pi r^{3}\varepsilon_{m}Re\left[K\left(\omega \right)\right]\nabla \left(\tilde{E}\cdot {\tilde{E}}^{*}\right)$$

where r is the cell radius, $\varepsilon_{m}$ is the medium permittivity, *E* is the electric field strength and $K\left(\omega \right)$ is the Clausius–Mossotti factor which is given by:

(4) $$K\left(\omega \right)=\frac{\varepsilon_{p}^{*}-\varepsilon_{m}^{*}}{\varepsilon_{p}^{*}+2\varepsilon_{m}^{*}}$$

(5) $$\varepsilon_{p}^{*}=\varepsilon_{p}-j\frac{\sigma_{p}}{\omega }$$

(6) $$\varepsilon_{m}^{*}=\varepsilon_{m}-j\frac{\sigma_{m}}{\omega }$$

where $\varepsilon_{p}$ and $\varepsilon_{m}$ are the permittivity of particles or cells and medium, $\sigma_{p}$ and $\sigma_{m}$ are the conductivity of particles or cells and medium and ω is the angular frequency of the signal. The calculation of yeast cells is based on a 2-shell spherical model. The calculation of K562 cells is based on a 1-shell spherical model. The electrical properties of yeast cells are based on a publication from Talary et al. [55]. The electrical properties of K562 cells are based on a publication from Demircan et al. [56].

The DEP velocity of particles and cells is defined as:

(7) $$<V_{DEP}>\;=\frac{<F_{DEP}>}{f_{r}}$$

where $f_{r}$ is the resistance factor of particles or cells.

## 3. Methods

### 3.1. Device Fabrication

A schematic of the fabrication process is given in Figure S1. The major fabrication steps include: (1)–(4) PDMS channel fabrication, (5)–(8) ITO electrode patterning and (9) plasma bonding. A photograph of our device with electrical connections and tubing is shown in Figure 1g. A microscopy image of our device is shown in Figure 1h. The PDMS microchannel, with a height of 70 μm and width of 540 μm, was fabricated by photolithography and PDMS replica molding. First, the channel mold was prepared by spin-coating a 70 μm thick layer of SU-8 2050 photoresist (MicroChem, Inc., Washington, MA, USA) in a substrate and then patterned by optical lithography. The channel mold needs to be treated with trichloro (1H,1H,2H,2H-perfluorooctyl) silane (97%, SigmaAldrich, Shanghai, China) for one hour to modify its surface. After that, a mixture of polydimethylsiloxane base and cross-linker (Sylgard 184, Dow Corning, Midland, MI, USA) at a ratio of 10:1 (*w*/*w*) was poured onto the mold and cured at 90 °C for 1 h. Finally, the channel was punched to open an inlet and an outlet for sample loading and unloading after the cured PDMS was peeled off. In order to have a clear observation of fluid and sample, a glass substrate which was coated with ITO film was used for fabricating electrodes. To fabricate the ITO electrode patterns, we first spin-coated the positive AZ4620 photoresist on the surface of ITO film, followed by optical lithography and developing. Lastly, the ITO film was etched by HCl solution to obtain electrode patterns. The parameters of electrodes are summarized in Figure S3 and Table S1. Once both PDMS channel and ITO electrodes were prepared, they were treated with oxygen plasma and then bonded together. The details of the fabrication process can be found in our previous work [40].

### 3.2. Sample Preparation

Buffer solution should have a low conductivity which can reduce the Joule heating effect. In this work, buffer solution was composed of 13% (*w*/*v*) sucrose + 2% (*w*/*v*) PBS + 85% (*w*/*v*) deionized water, which had a conductivity of 19 mS/m. The samples used in our study were PS microbeads with diameters of 10 and 15 μm, yeasts cells and K562 cells. To prepare yeast cells, 50 mg of baker’s dry yeast was reactivated in 10 mL of DI water at 30 °C for 1 h. After that, yeast suspension of 1.5 mL was transferred to a centrifuge tube and washed three times. Then, the yeast cells were resuspended in 8 mS/m KCl solution. PS microbeads were transferred to KCl solutions with σ_m_ = 8 mS/m to make a suspension for the experiments. To obtain the required cell or particle density, the yeast and microbead solutions were diluted with KCl solution of the same conductivity. Human myelogenous leukemia cell line K562 was grown at 37 °C, 5% (*v*/*v*) CO_2_. The culture medium consisted of RPMI 1640 (Biological Industries), supplemented with 10% (*v*/*v*) heat-inactivated fetal bovine serum (FBS) and 1% (*v*/*v*) penicillin–streptomycin (Biological Industries). Before the experiment, the cells were incubated with 0.1 mg/mL fluorescein diacetate (FDA) (Sigma-Aldrich) in culture medium (37 °C, 5 min). Then, they were washed three times with buffer solution. To obtain the required cell density, the K562 cell solutions were diluted with buffer solution of the same conductivity.

### 3.3. Experimental Setup

The AC signals energized on the ITO electrodes were generated by using a function generator (DG4062, RIGOL, Beijing, China). Samples were stably injected into the microchannel by a pump (Wenhao Co., Ltd., Suzhou, China). Images and videos were observed using a microscope (NIB-100, Novel Optics, Ningbo, China) with a CCD camera (T830, Novel Optics, China). All acquired images and videos were analyzed by ImageJ.

## 4. Results and Discussion

### 4.1. Rotating Mechanism of the ICEO Method

**Figure 2:**
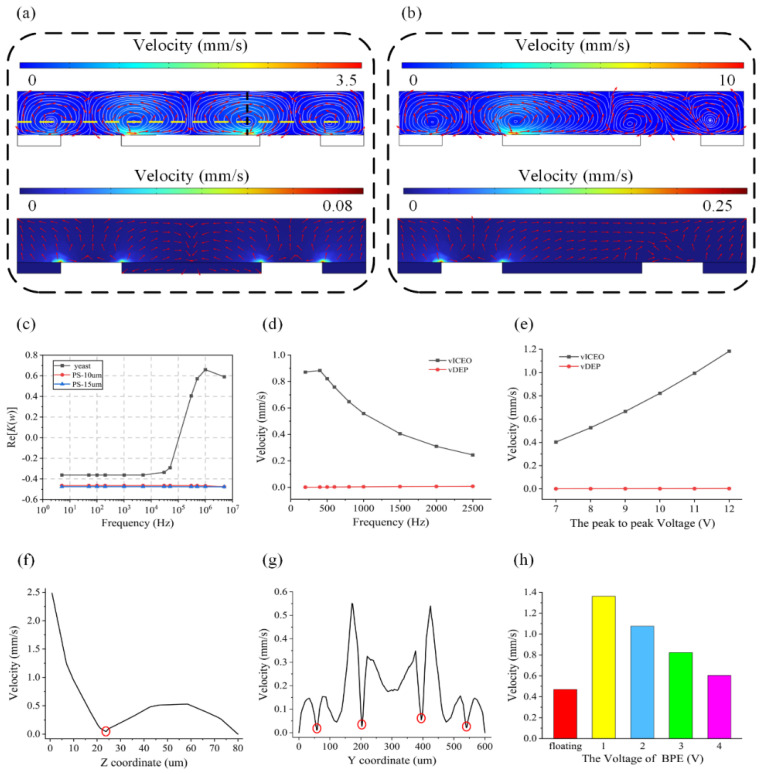
(**a**) The distribution of ICEO velocity (top) and DEP velocity (bottom) in the microchannel under an AC field of V = 10 V, V_g_ = floating and f = 500 Hz. There are two vortexes above the center electrode which are symmetrical and rotating in opposite directions. Another two vortexes are respectively above the driving electrodes. They rotate in opposite directions symmetrically. Red and blue colors indicate high and low velocity of ICEO (top) and DEP (bottom), respectively. The direction of ICEO (top) and DEP (bottom) streaming lines is indicated by the red arrows. Yellow and black dashed lines show the position of the vortex center. (**b**) The distribution of ICEO velocity in the microchannel under an AC field of *V* = 10 V, *V*_g_ = 1 V and *f* = 500 Hz. The rotation directions of four vortexes are the same as in (**a**), but they become asymmetrical. (**c**) Real parts of the CM factors as a function of frequency for yeasts, 10 μm PS particles and 15 μm PS particles at suspending medium conductivity of 8 mS/m. (**d**) Frequency-dependent electrode surface average velocity of ICEO and DEP of yeasts under an AC field of V = 10 V and V_g_ = floating. (**e**) Voltage-dependent electrode surface average velocity of ICEO and DEP of yeasts under an AC field of V_g_ = floating and f = 500 Hz. (**f**) ICEO velocity of y–z plane at black dashed line in (**a**). (**g**) ICEO velocity of y-z plane at yellow dashed line in (**a**). (**h**) Relationship between the average velocity of ICEO at surfaces of left driving electrode and the applied voltages of V_g_ under an AC field of V = 10 V and f = 500 Hz.

To understand the role of the DEP force and ICEO flow on the cell rotation, we first simulated the distribution of ICEO velocity and DEP velocity on the y–z plane of the PDMS channel using a commercial software package (COMSOL Multiphysics 6.0). The electric current interface and the laminar flow interface [57,58] were used here. Equation (S4) or (S6) was the boundary condition of the center electrode under different circumstances. For the boundary condition of the driving electrode, Equation (S6) can be a reference. By using those boundary conditions, we can simulate the ICEO in COMSOL. Under an AC electric field of V = 10 V, V_g_ = floating and f = 500 Hz, the simulation results indicate that the ICEO flow dominates over the DEP velocity as shown in Figure 2a, since the ICEO velocity in the channel is much faster than the DEP velocity. The ICEO flow will maintain its dominance when the frequency changes from 200 Hz to 2500 Hz or the amplitude changes from 7 V to 12 V (Figure 2d,e). When the ICEO-induced drag force is powerful enough to balance the gravitational force and DEP force, the cell floats to the equilibrium position in the center of the microrecirculation zone. The cell is then captured at the center of the microvortex and the shear stress from the recirculation flow in the microvortex induces the out of plane rotation of the cell located in the vortex. The steady ICEO microvortexes formed above the electrodes can be used to rotate cells precisely. As shown in Figure 2a (top row), the ICEO produces four symmetric vortexes in the microchannel which rotate, respectively, clockwise, counterclockwise, clockwise and counterclockwise. It is noted that two of the vortexes are located above the center electrode and the other two vortexes occur above the driving electrodes. In addition, the centers of those vortexes are not on the surface of electrode edges but above the surfaces and near the edges. To identify the position of the vortex center, we simulate the ICEO velocity of the y–z plane along the yellow and black dashed lines in Figure 2a. Figure 2f shows the velocity distribution on the black dashed line, where there is a minimum point which is at about 24 μm on the z-axis marked with a red circle. We take this point as a reference to draw the yellow dashed line and plot the relationship between the ICEO velocity and Y coordinate in Figure 2g. Except for the gaps between electrodes and the walls of the channel, we find four points which are marked with red circles that have lower velocity. We believe that those four points are the approximate locations of the vortex centers as vortex centers have the minimum ICEO velocity where the cell will be trapped. Y coordinates of those four points are about 57, 203, 395 and 541 μm, while electrode edges’ Y coordinates are 75, 180, 420 and 525 μm. Figure 2c shows a plot of the real parts of Clausius–Mossotti factors for yeast cells and PS particles at the suspending medium conductivity of 8 mS/m. When the frequency is lower than 100 kHz, both yeast cells and PS particles are influenced by nDEP force which can help them to move away from the surface of electrodes and be trapped in a vortex. To explore the ability of this method for adjusting the position and direction of cell rotation, when we further apply a voltage of 1 V to the center electrode, the zeta potential distribution above the center electrode will be modified which leads to the occurrence of asymmetric vortexes across the channel for changing cell rotation position. It is noted that the ICEO velocity on the left side of the channel will be enhanced due to the asymmetric distribution of zeta potential (Figure 2b). Moreover, we analyze the relationship between the average velocity of ICEO at surfaces of the left driving electrode and the applied voltages of V_g_ (Figure 2h). The simulation results indicate that the average velocity of ICEO at surfaces of the left driving electrode is much faster when V_g_ < V/2 which will help to decrease the applied power.

### 4.2. Controllable Rotation of Yeast Cells

**Figure 3:**
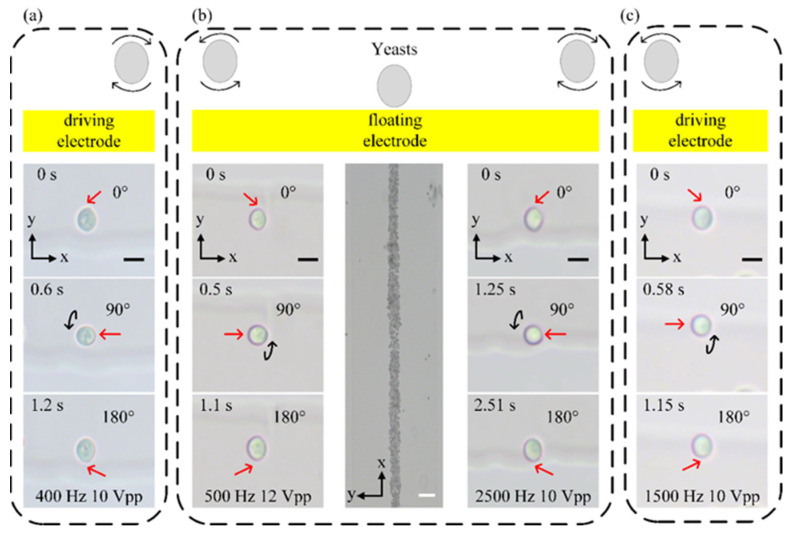
Schematic of the device in operation. (**a**) Schematic and image sequences showing that the yeast is continuously rotated clockwise at up driving electrode (Movie 1). (**b**) Schematic and image sequences showing that the yeast is continuously rotated counterclockwise at top edge of floating electrode, clockwise at bottom edge of floating electrode. Some yeasts are focused in the middle of floating electrode (Movie 2). (**c**) Schematic and image sequences showing that the yeast is continuously rotated counterclockwise at down driving electrode (Movie 1). Red arrows indicate a specific point on yeast cells. Black scale bar, 5 μm. White scale bar, 100 μm.

**Figure 4:**
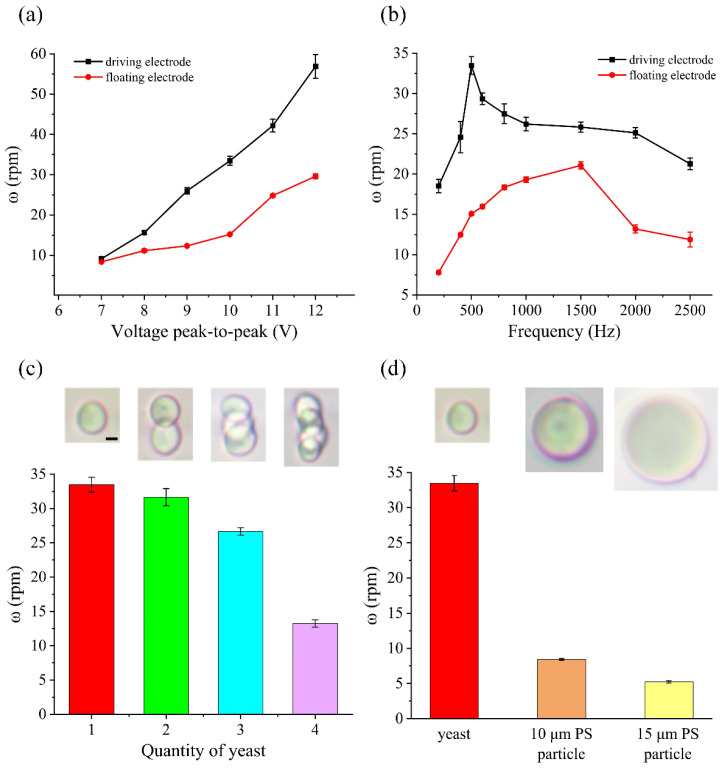
(**a**) Plot of the rotation speed against voltage of a yeast cell under an AC field of V_g_ = floating and f = 500 Hz. (**b**) Plot of the rotation speed against frequency of a yeast cell under an AC field of V = 10 Vpp and V_g_ = floating. (**c**) Plot of the rotation speed against quantity of yeast under an AC field of V = 10 Vpp, V_g_ = floating and f = 500 Hz. (**d**) Plot of the rotation speed against diameter of manipulated object under an AC field of V = 10 Vpp, V_g_ = floating and f = 500 Hz. Scale bar, 2 μm.

Cell rotations are important in determining cell morphology accurately by cell imaging and performing cell surgery. To have a good observation, the rotation speed of cells cannot be too fast due to the limited observation capabilities of the human eye. On the other hand, it will increase the detection time if the rotation speed is too slow. In addition, since our method can rotate cells in a precise and controllable manner, we can further rotate different types of cells with various sizes at a fixed speed based on the ICEO flow. To demonstrate the rotation performance of our method, we conducted rotation experiments using yeast cells. As shown in Figure 3, when an electric field was applied (*Vg* = floating), under nDEP force and ICEO, yeast cells moved from electrode edges to centers and from electrode surfaces to above the electrode. Then, yeast cells were trapped by vortexes induced by ICEO vortexes (Figure 3a, 3b (left row), 3b (right row) and 3c) and continuously rotated or focused into a particle beam in the middle of the center electrode (Figure 3b (middle row)). The rotation directions of yeast cells were different depending on the position. Yeast cells rotated clockwise in the up driving electrode and bottom edge of the center electrode (Figure 3a and 3b (right row)) and rotated counterclockwise in the down driving electrode and top edge of the center electrode (Figure 3b (left row) and 3c). Furthermore, the rotation speed of yeast cells can be changed depending on the applied signals and the rotation position. We further analyzed and characterized the rotation speed of yeast under different conditions. We first investigated the influence of the applied voltage on the rotation performance, as shown in Figure 4a. At a frequency of 500 Hz, when the applied voltage increases, the yeast cells rotate faster. Moreover, the rotation speeds of yeast cells at driving electrodes were faster than that at the floating electrode. When the voltage was 7 V_pp_, yeast cells were rotated at about 9.1 rpm near the driving electrode and at about 8.3 rpm near the floating electrode. As the voltage increased to 12 V_pp_, the rotation speed was increased to about 56.8 rpm at the driving electrode and reached about 29.6 rpm at the floating electrode. Compared with the simulation results (Figure 2e), the rotation speed of yeast cells changes with voltage in the same trend as the ICEO speed. This is also consistent with (S8). The slip velocity is positively related to the electric field strength. The higher slip velocity creates vortexes with faster speed that rotate yeast cells faster. Subsequently, we studied the effect of the applied frequency on the rotation performance at a fixed voltage of 10 V_pp_, shown in Figure 4b. It is clear that the speeds of yeast cells at the driving electrodes were faster than that at the floating electrode. For yeast cells at the driving electrode, the rotation speed was about 18.5 rpm under a frequency of 200 Hz, increased to 33.4 rpm at 500 Hz and finally decreased to 21.2 rpm under a frequency of 2500 Hz. For yeast at the floating electrode, the rotation speed was about 7.7 rpm under a frequency of 200 Hz, increased to 21.1 rpm at 1500 Hz and decreased to 11.8 rpm under a frequency of 2500 Hz. It is noted that the rotation performances at the floating and driving electrodes show the same change tendency when applying different frequencies. Although there is a slight difference between the frequency corresponding to the peak rotation speed and the simulation results (Figure 2d), they all conform to the ICEO theory [30] that ICEO velocity will increase with frequency as the frequency is below the characteristic RC charging frequency. ICEO flow decays rapidly above the characteristic RC charging frequency due to a relaxation process. Apart from the above factors, we also investigated the relationship between the rotation speed and the diameter of the manipulated object. Generally, the diameter of yeast is 5 μm, and sometimes yeast cells aggregate together which makes them bigger. So, we selected clusters which had different numbers of yeast cells and PS particles with diameter of 10 μm and 15 μm to calculate their speed under the same conditions. As shown in Figure 4c,d, the rotational speed of the sample decreased as the diameter of the manipulated object increased. Overall, our device could not only rotate yeast cells simultaneously in the y–z plane, which would provide a comprehensive view of cells without a confocal microscope, but also allow the rotation of cells with different sizes by selecting an appropriate voltage and frequency.

### 4.3. Controllable Rotation of K562 Cells

**Figure 5:**
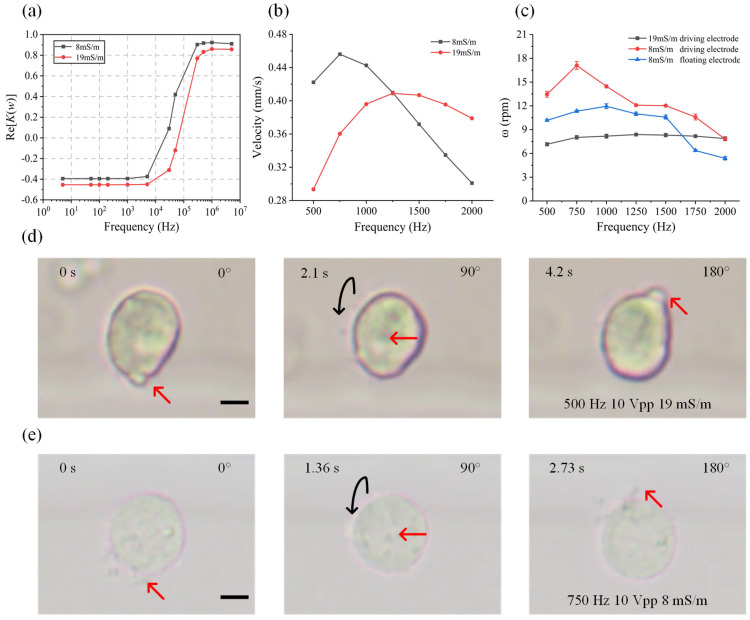
(**a**) Real parts of the CM factors as a function of frequency for K562 cells at suspending medium conductivities of 8 mS/m, 19 mS/m. (**b**) Frequency-dependent driving electrode surface average velocity of ICEO of K562 cells under an AC field of V = 10 Vpp and V_g_ = floating. (**c**) Plot of the rotation speed against frequency under an AC field of V = 10 Vpp, V_g_ = floating. (**d**,**e**) Image sequence showing that the K562 cell is continuously rotated clockwise at suspending medium conductivities of 19 mS/m (Movie 3), 8 mS/m. Red arrows indicate a specific point on K562 cells and black arrows show the rotation direction. Scale bar, 3 μm.

In the aforementioned experiments, we achieved a three-dimensional rotation of yeast, a representative fungus. To demonstrate the applicability and universality of our method, we select K562 cells as a representative of biological cells to carry out the experiments. K562 cells are the first artificially cultured cells of human myeloid leukemia, which are widely used in the research of tumor and leukemia therapy, drug targets and other fields. Here, we conducted the rotation experiments using K562 cells to verify the versatility of our method. The K562 cell solutions were diluted with buffer solution at both 8 mS/m and 19 mS/m to investigate the influence of the medium conductivity on the rotation performance. Firstly, we simulated the real parts of the CM factors of K562 cells as a function of the applied frequency in aqueous solutions with different medium conductivities (Figure 5a). The frequency-dependent surface averaged ICEO velocity at the driving electrode surface is displayed in Figure 5b. The simulation results show that K562 cells experience an nDEP force at a low frequency, while the averaged ICEO velocities on the driving electrode surfaces increase first and then decrease with frequency in solutions with different conductivities. When the applied frequency is less than 1250 Hz, the rotation speed of the K562 cell in a low-conductivity medium with 8 mS/m is faster than that in aqueous solutions with 19 mS/m. The experimental results are consistent with the simulation data. As shown in Figure 5c, for K562 cells in aqueous solutions with a conductivity of 8 mS/m, the rotation speed near the driving electrode is about 13.4 rpm under a frequency of 500 Hz, increases to a peak of 17 rpm at 750 Hz and then decreases to 7.7 rpm under a frequency of 2000 Hz. For K562 cells near the floating electrode, the rotation speed was about 10.1 rpm under a frequency of 500 Hz, increased to a peak of 11.9 rpm at 1000 Hz and then decreased to 5.3 rpm under a frequency of 2000 Hz. In aqueous solutions with a conductivity of 19 mS/m, the rotation speed of K562 cells located near the driving electrode was about 7.1 rpm under a frequency of 500 Hz, increased to a peak of 8.4 rpm at 1250 Hz and then decreased to 7.8 rpm under a frequency of 2000 Hz. The image sequence of K562 cells is shown in Figure 5d,e. It is noted that the rotation speed of K562 cells at the driving electrode was faster than that at the floating electrode. The rotation speed of K562 cells in aqueous solutions with 8 mS/m was faster than that in solutions with 19 mS/m when the applied frequency increased to 2000 Hz. Furthermore, we studied the influence of ICEO rotation on the cell viability using FDA in the experiments. K562 cells were observed and compared before and after the rotational manipulation of cells. All cells remained green under fluorescence after a two-hour experiment (Figure S2), which demonstrates that our proposed rotation method is safe for cells.

### 4.4. Bidirectional Rotation of Cells

**Figure 6:**
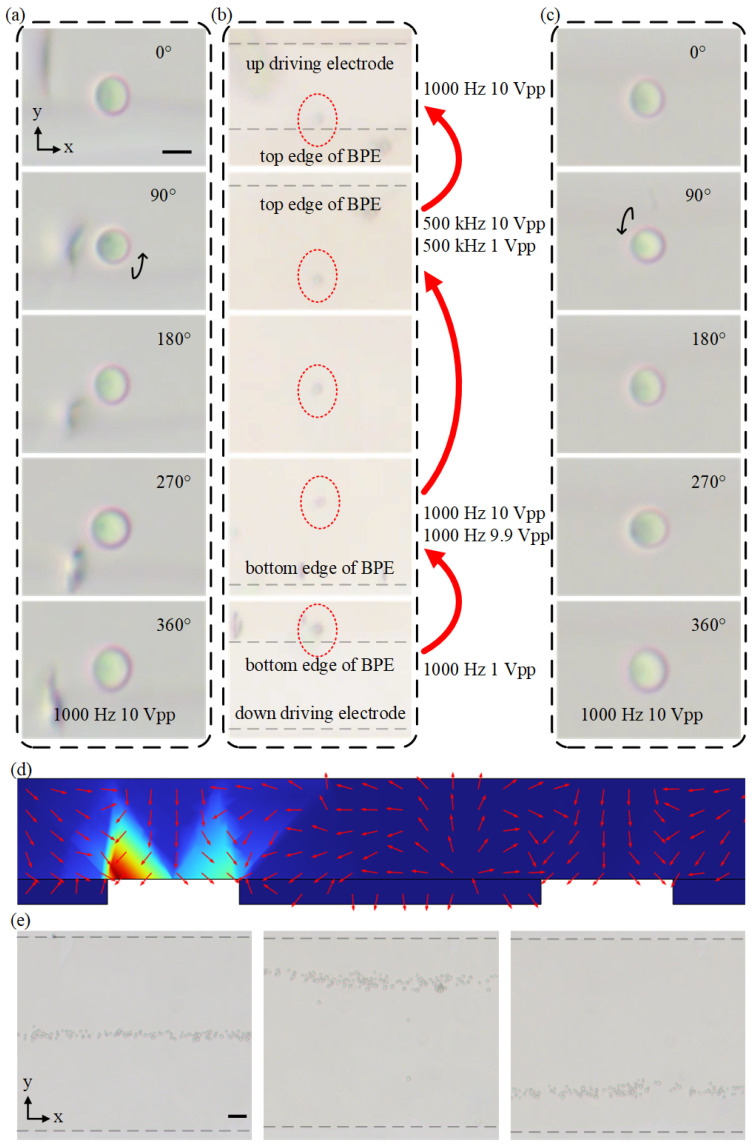
Rotating yeast cells in opposite directions using ICEO. (**a**) Image sequences showing that the yeast is continuously rotated counterclockwise at the bottom edge of BPE. Black arrow displays the rotation direction. (**b**) Image sequences showing the process of moving yeast rotating at the bottom edge of BPE to the top edge of BPE. The red circles display the yeast cells. (**c**) Image sequences showing that the yeast cell is continuously rotated clockwise at the top edge of BPE (Movie 4). (**d**) The simulation results of DEP velocity distribution in the microchannel under an AC field of V = 10 V, V_g_ = 1 V and f = 500 kHz. The direction of DEP streaming lines is indicated by the red arrows. Scale bar, 5 μm. (**e**) Image of yeasts focused in the middle of center electrode (left panel), at the top edge of center electrode (center panel) and at the bottom edge of center electrode (right panel). Scale bar, 50 μm.

When the center electrode is floating, four symmetrical ICEO vortexes occur and the cells can be trapped and rotated in the microvortexes. To exploit the ability of adjusting the direction of cell rotation, we further investigate the effect of the gate voltage applied to the center or bipolar electrode (BPE) on the rotation position and direction. When a gate voltage is applied to the center electrode, asymmetric vortexes can be produced in the microchannel, which can be used to adjust the cell-trapping position due to the location change of the vortex center. The detailed process for adjusting cell-rotating position is shown in Figure 6e. When the center electrode is floating, the yeast cells are trapped in the middle of the center electrode and form a focused particle beam (Figure 6e). Then, by applying V_g_ = 9.5 Vpp or V_g_ = 0.5 Vpp (V = 10 Vpp), we could either focus the yeasts at the top edge of the center electrode or the bottom edge of the center electrode. Thus, via adjusting the BPE voltage, the position of cells can be changed which will allow the bidirectional rotation of yeast cells. Figure 6 shows the experimental process of rotating yeast cells in opposite directions by changing the cell-rotating position. A yeast cell was rotated counterclockwise at the bottom edge of the center electrode under a signal of 1000 Hz and 10 Vpp (Figure 6a). When the applied voltage becomes 1 Vpp, the ICEO velocity decreases quickly and the yeast cells fall down to the electrode surface due to gravity. After yeast cells sink to the surface of the electrode, a signal of V = 10 Vpp and Vg = 9.9 Vpp is applied and large ICEO flow vortexes appear at the bottom edge of the center electrode. The large vortexes would push yeast cells away and, as a result, cells are unable to be trapped in the vortexes. The yeast gradually slowed down as it slid from the bottom edge to the top edge. Afterward, we applied a signal of f = 500 kHz and V_g_ = 1 Vpp. At this time, yeast cells experience pDEP force and are attracted toward the top edge of the center electrode (Figure 6b). The signal applied at the center electrode would generate an asymmetric pDEP effect which could enhance the pDEP force of yeast near the top edge (Figure 6d). As soon as the yeast was trapped at the top edge of the center electrode, we applied a signal of 1000 Hz 10 Vpp again to rotate the yeast clockwise at the top edge of the center electrode (Figure 6c). Apart from moving cells at the center electrode and changing the direction of rotation, asymmetric vortexes also could augment the rotation speed of cells at one side of the channel. The rotation speed of yeast cells under the condition of a 0.5 Vpp signal at the center electrode and a 5 Vpp signal at the driving electrode is close to the speed with a 10 Vpp signal at the driving electrode. This would decrease the voltage to rotate cells and reduce consumption.

### 4.5. Stepwise Rotation of Cells

**Figure 7:**
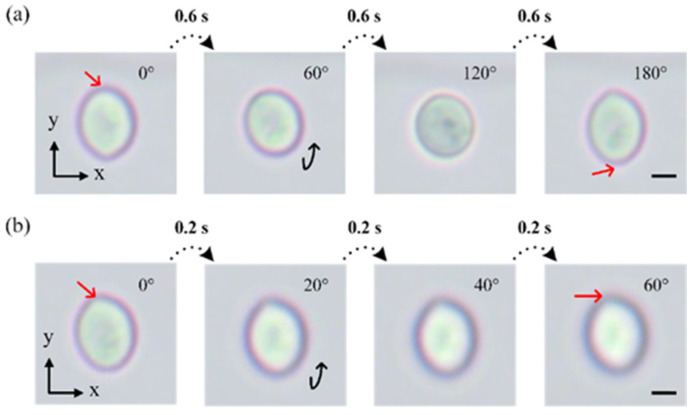
Image sequences showing the stepwise rotation of yeasts when supplied with 500 Hz 10 Vpp pulsed signals of (**a**) 0.6 s duration and (**b**) 0.2 s duration (Movie 5). Red arrows indicate a specific point on cells Scale bar, 2 μm.

In cell research, sometimes we need to observe a specific location of cells which requires highly precise and controllable manipulation. By applying a pulsed signal instead of a continuous signal, we further exploit the stepwise rotation of cells in a controllable manner using our proposed device. Cells can be rotated stepwise at any angle we needed. For example, yeast cells would rotate by 60° when a signal pulse of 500 Hz and 10 Vpp with a duration of 0.6 s is applied. After three steps, yeast was rotated by 180° (Figure 7a). By reducing the duration of the pulse down to 0.2 s, the same yeast cell could be rotated by 20° in one step (Figure 7b). According to the results in Figure 4a,b, the stepwise rotation angle can also be flexibly adjusted by changing the applied voltage and frequency. In the process of step rotation, yeast cells did not drift along the channel apparently and cell viability is unaffected (Figure S2). Thus, with the above stepwise manipulation using our technique, the views of cells from any angle can be controlled and observed for further analysis.

We also summarized and compared some reported electric methods and our proposed device as shown in Table 1 below. Our method ensures all kinds of particles or cells can be rotated regardless of their properties based on ICEO flow. In contrast with other electric methods, we proposed a microfluidic device to rotate cells and particles in 3D. The rotation speed can be controlled by adjusting the voltage and frequency of the applied signals. By flexibly adjusting the applied signals at driving electrodes and the center electrode, cells can be moved from one vortex to another and the rotation direction will be changed as well. Additionally, in our methods, cells can be rotated stepwise by applying a pulsed signal. The rotational manipulation of this ICEO-based method does not rely on physical properties of the cells and particles which makes this method universal. However, the electrode may be electrolyzed when the amplitude of the applied signal is too high. For example, when the frequency of the applied signal is 500 Hz, 20 V will be the upper limit. In addition, the stability of rotation is determined by the signals applied. From the movie, we can see the slight change of yeast cells’ position in both x- and y-directions, although the rotation of K562 cells is more stable. Although the positions of yeast cells were slightly changed during the rotation process, the rotation process can still be controlled precisely and the rotation performance can be evaluated accurately. In addition, the performance of cell rotation can also be further improved by optimizing and adjusting the medium conductivity, applied signal and the electrode design.

## 5. Conclusions

In this paper, we for the first time proposed an ICEO-based method for precise rotation of different cells, including yeast cells and K562 cells. The rotation speeds of cells are controlled by voltage, frequency and solution conductivity. By applying a gate voltage to form the asymmetric ICEO microvortexes, the rotation position of cells can be flexibly adjusted which further changes the rotation direction of cells. Moreover, our device can not only rotate cells continuously but it can also rotate cells stepwise by applying a pulsed signal. Yeast cells would rotate by 60° when a pulsed signal of 500 Hz and 10 Vpp with a duration of 0.6 s is applied. The angle of stepwise rotation can be adjusted by changing the duration of the pulsed signal. In contrast to mere DEP-based methods, using this proposed hybrid method based on ICEO flow, multiple cells regardless of their properties can be rotated by adjusting the ICEO flow via changing the applied electric signal. These results verified the unique ability of this method to guide and control the rotation of living materials in an ordered and predetermined way. Our approach is simple to fabricate and operate and can be extended to other cells or small organisms by design modifications of devices. It can also be integrated with other devices to compose platforms for bioengineering, biophysics and medicine studies.

## Figures and Tables

**Table 1:** An overview of recently reported electric methods for rotating cells.

Method	Rotation Stability	Change Direction	Stepwise	Electric Property
Habaza et al. [1]	High	Yes	No	Related
Huang et al. [19]	High	Yes	No	Related
Benhal et al. [20]	High	Yes	No	Related
Puttaswamy et al. [29]	High	No	No	Related
Our work	Normal	Yes	Yes	Unrelated

## Data Availability

Data are contained within the article.

## Supplementary Materials

The following supporting information can be downloaded at: https://www.mdpi.com/article/10.3390/bios14030112/s1, Figure S1: A schematic of the fabrication process of the microfluidic device including: (1)–(4) PDMS channel fabrication, (5)–(8) ITO electrodes patterning, and (9) plasma bonding; Figure S2: The cell viability of K562 (a) and yeast (b) cells in the experiment. All cells remain green under fluorescence; Figure S3: A schematic of the electrodes and channel of the microfluidic chip; Figure S4: The rotation speed of yeast cells in different conditions. Green: a signal of 0.5 Vpp, 400 Hz at the center electrode and a signal of 5 Vpp, 400 Hz at the driving electrode. Blue: a signal of 10 Vpp, 400 Hz at the driving electrode; Table S1: Geometrical parameters for the structure of the proposed chip.
