# Supplementary material for: Bidirectional and Stepwise Rotation of Cells and Particles Using Induced Charge Electroosmosis Vortexes
Source: Biosensors (Basel). 2024 Feb 20;14(3):112. doi: 10.3390/bios14030112 (PMC10968096; doi:10.3390/bios14030112)
Supplement: Supplementary file 1 [file biosensors-14-00112-s001.zip › biosensors-2849434-supplementary.pdf]

1. Theoretical Background

A. Electric Field

The electrical potential in the bulk electrolyte can be calculated by solving Laplace's equation, assuming the bulk concentration is homogeneous.

$$\nabla \cdot (\sigma E) = -\sigma \nabla^2 \phi = 0 \quad \text{(S1)}$$

where E is the electric field, ϕ is the bulk potential and σ is the liquid conductivity.

The Laplace equation is subjected to the boundary conditions as follows:

At the driving electrodes, the boundary conditions are shown as follows:

One: $\phi(t) = V\cos(\omega t)$, and the other ground: $\phi(t) = 0$ (S2a)

At the insulating surface, the boundary condition can be simplified to

$$\frac{\partial \phi}{\partial y} = 0 \quad \text{(S2b)}$$

The double layer on the electrode describes the charging of the induced double layer owing to the current in the bulk. In the case of low voltage across the diffuse layer, the surface conservation equation is:

$$C_D \frac{d\zeta}{dt} = C_0 \frac{d\Psi_0}{dt} = \frac{d(\phi_g - \phi)}{dt} \frac{C_D}{1+\delta} = -\sigma \hat{n} \cdot \nabla \phi = \sigma E_n \quad \text{(S2c)}$$

where $\zeta = V_g \cos(\omega t + \theta_g) - \phi(t)$ is the zeta potential, θ_g is the phase gap in the applied gate potential with reference to the driving signal. V and V_g are the voltage amplitude applied to the driving electrode and center electrode, respectively. $\phi(t)$ is the potential in the bulk just outside the double layer. $C_0 = \frac{C_S C_D}{C_S + C_D} = \frac{C_D}{1+\delta}$ is the capacitance per unit of area of the whole induced double layer, the parameter $\delta = C_D / C_S$ is the ratio of the diffuse layer capacitance ($C_D = \varepsilon / \lambda_D$) to Stern layer capacitance (C_S), ε is the permittivity, $\lambda_D = \sqrt{D\varepsilon/\sigma}$ is the Debye screening length, $D = 2 \times 10^{-9}\ \mathrm{m^2\ s^{-1}}$ is the bulk diffusivity and $\varepsilon = 7.08 \times 10^{-10}\ \mathrm{F\ m^{-1}}$ is the permittivity.

As for fixed potential ICEO, an analytical solution for the induced zeta potential in the DC limit can be achieved as follows.

(1) When the center electrode is floating, the induced zeta potential is

$$\zeta(t) = \frac{1}{1+\delta}\left(\frac{V}{2}\cos(\omega t) - \phi(t)\right) = \frac{1}{1+\delta} Ex\cos(\omega t) \quad \text{(S3)}$$

The ICEO slip velocity on the surface of the floating electrode is given by the Helmholtz–Smoluchowski equation:

$$\langle v_s \rangle = \frac{-\varepsilon}{\eta} \langle \frac{1}{1+\delta} Ex\cos(\omega t) \cdot E_t \rangle = \frac{-\varepsilon E^2 x}{2\eta(1+\delta)} \quad \text{(S4)}$$

(2) Assuming the phase gap $\theta_g = 0$, when an electric signal $V_g = \cos(\omega t)$ is applied to the center electrode, the zeta potential becomes:

$$\zeta(t) = \frac{1}{1+\delta}\left(V_g \cos(\omega t) - \phi(t)\right) = \frac{1}{1+\delta}\left(Ex\cos(\omega t) + (V_g - \frac{V}{2})\cos(\omega t)\right) \quad \text{(S5)}$$

The ICEO slip expression becomes:

$$\langle v_s \rangle = \frac{-\varepsilon}{\eta} \langle \frac{1}{1+\delta}(Ex + V_g - \frac{V}{2})\cos(\omega t) \cdot E_t \rangle = \frac{-\varepsilon E^2 x}{2\eta(1+\delta)}(Ex + V_g - \frac{V}{2}) \quad \text{(S6)}$$

The S4 or S6 will be the boundary condition of the center electrode under different circumstances. For the boundary condition of the driving electrode, (S6) can be a reference. By using those boundary conditions, we can simulate the ICEO in COMSOL.

The flow stagnation line (FSL) can be achieved by making $\langle v_s \rangle$ equal to zero. It can be seen that the FSL is located at the center of the floating electrode ($x = 0$) when the center electrode is set as floating. The FSL can be changed ($x = \frac{1}{E}(\frac{V}{2} - V_g)$) by altering the electric signal. For instance, when the center electrode is energized with $V_g \cos(\omega t)$ ($V_g > V/2$),

positive charges will be accumulated at the top surface of the center electrode, resulting in the formation of a negatively charged double layer in the liquid above the center electrode. Consequently, an asymmetric ICEO flow occurs and FSL is directed away from the ground electrode. On the other hand, when $V_g < V/2$, a positively charged double layer forms and FSL is directed toward the ground electrode. Equations (S5) and (S6) show that altering the electric potential applied to the center electrode will change the zeta potential above the electrode, resulting in shifting the flow stagnation line and generating asymmetric flow across the channel. The direction of the asymmetry can also be reversed by changing the potential of the center electrode.

Flow Field

For a Newtonian incompressible flow, the liquid motion can be obtained by solving the Navier–Stokes equations and the continuity equation in the limit of a small Reynolds number.

$$\rho\left(\frac{\partial \bar{v}}{\partial t}\right) = \eta\nabla^2\bar{v} - \nabla p,\ \ \nabla\bar{v} = 0 \tag{S7}$$

p is the pressure, $\bar{v}$ is the velocity.

For thin double layers and weak fields, the time-averaged slip velocity at the interface between the bulk and the double layer can be derived from the Helmholtz–Smoluchowski formula [1].

$$\langle v_s\rangle = \frac{-\varepsilon}{\eta}\frac{1}{2}\mathrm{Re}(\tilde{\zeta}\widetilde{E_t}^{*}) = \frac{-\varepsilon}{\eta}\frac{1}{1+\delta}\frac{1}{2}\mathrm{Re}((\widetilde{\phi_g} - \tilde{\phi})(\tilde{E} - \tilde{E}\cdot n\cdot n)^{*}) \tag{S8}$$

where E_t is the tangential electric field, ζ is the zeta potential, η is the viscosity of the fluid, the asterisk indicates the complex conjugate. Although some equilibrium potential may exist on the channel walls, it can be safely neglected here due to linear electroosmotic slip, whose time-averaged value is zero in an AC field. All other surfaces should be treated as no-slip walls.

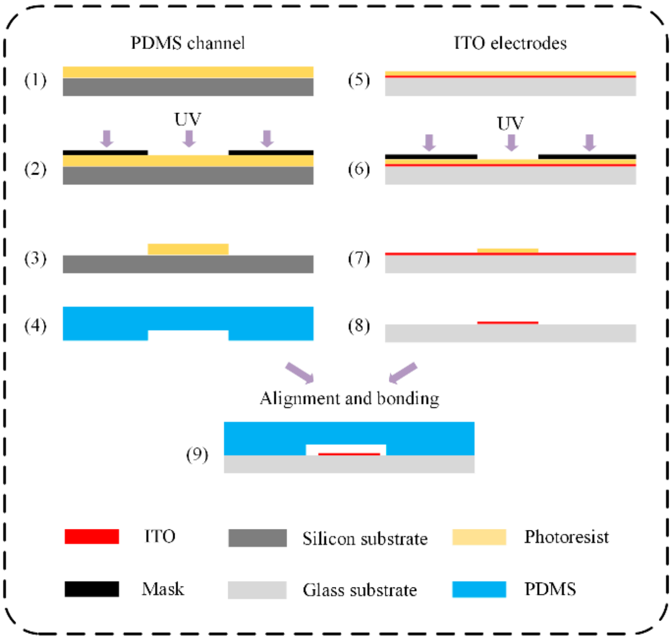

Figure S1. A schematic of the fabrication process of the microfluidic device including: (1)–(4) PDMS channel fabrication, (5)–(8) ITO electrode patterning and (9) plasma bonding.

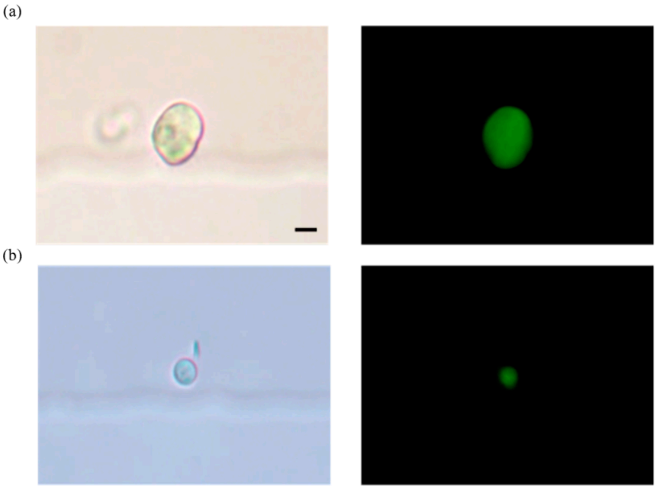

Figure S2. The cell viability of K562 (a) and yeast (b) cells in the experiment. All cells remain green under fluorescence.

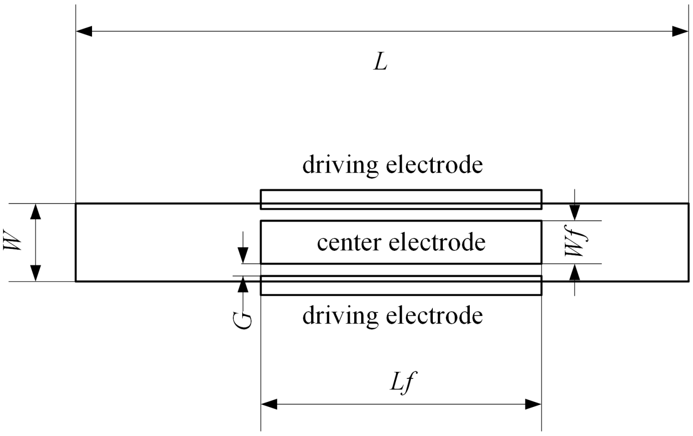

Figure S3. A schematic of the electrodes and channel of the microfluidic chip.

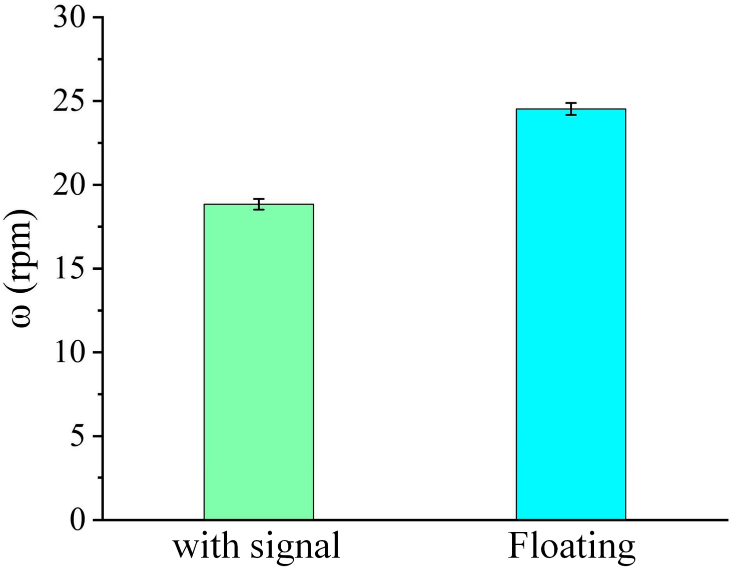

Figure S4. The rotation speed of yeast cells in different conditions. Green: a signal of 0.5 Vpp, 400 Hz at the center electrode and a signal of 5 Vpp, 400 Hz at the driving electrode. Blue: a signal of 10 Vpp, 400 Hz at the driving electrode.

Table S1. Geometrical parameters for the structure of the proposed chip.

Parameters	Value (μm)	Implication
W	540	width of the channel
L	5000	length of the channel
H	70	height of the channel
G	50	the gap of the driving electrode to the center electrode
Wf	350	width of the center electrode
Lf	2290	length of the driving electrode

[1] Bazant, M.Z.; Squires, T.M. Induced-charge electrokinetic phenomena: Theory and microfluidic applications. *Phys. Rev. Lett.* **2004**, *92*, 066101. https://doi.org/10.1103/PhysRevLett.92.066101.
